# The influence of personality, alexithymia and work engagement on burnout among village doctors in China: a cross-sectional study

**DOI:** 10.1186/s12889-021-11544-8

**Published:** 2021-08-04

**Authors:** Xuewen Zhang, Xue Bai, Liyan Bian, Min Wang

**Affiliations:** 1 School of Integrated Traditional Chinese and Western Medicine, Jining Medical University, Jining, 272067 China; 2 School of the First Clinical Medicine, Jining Medical University, Jining, 272067 China

**Keywords:** Rural health human resource, Village doctors, Personality, Alexithymia, Work engagement, Burnout

## Abstract

**Background:**

In China, as the “gatekeepers”of rural residents’ health, the primary-level village doctors, play a very crucial role in ensuring and serving the health level of rural residents. However, the burnout of village doctors is gravely threatening the stability of rural primary medical system step by step. This study systematically evaluated the effects of personality, work engagement and alexithymia on burnout of village doctors, and further measured and assessed the mediating effect of alexithymia and work engagement in the association between personality and burnout.

**Methods:**

The subjects were 2684 village doctors in Jining, Shandong Province, China, from May to June 2019. Sociodemographic characteristics, alexithymia, personality, work engagement and job burnout were quantitated by self-completed questionnaire and measured by Likert 5–7 scale. One-way ANOVA, Person correlation analysis, and Structural Equation Modelling (SEM) were used for statistical analysis and mediating effect evaluation.

**Results:**

2693 questionnaires were collected in total, of which 2684 were valid, with an effective rate of 96.2%. 65.2% of village doctors were diagnosed with burnout, and 54.3% showed moderate to severe emotional exhaustion, 61.6% showed moderate to severe low sense of personal achievement, and 33.9% showed moderate to severe depersonalization burnout. Personality had a direct positive effect on work engagement (β = 0.50, *p* < 0.001), a direct negative effect on alexithymia (β = − 0.52, *p* < 0.001) and burnout (β = − 0.50, p < 0.001) respectively. Work engagement had a direct negative effect on burnout (β = − 0.10, p < 0.001), while alexithymia had a direct positive effect on burnout (β = 0.16, p < 0.001). In the path between personality and burnout, both work engagement 95%CI:(− 0.17)–(− 0.08), and alexithymia 95%CI:(− 0.36)–(− 0.09), have significant mediating effects. These results strongly confirm that personality, alexithymia, and work engagement are early and powerful predicators of burnout.

**Conclusion:**

According to the results, medical administrators should pay attention to the personality characteristics of village doctors in vocational training, practice selection and job assignment, encourage village doctors to reflect on their own personality actively, and to reduce job burnout by obtaining necessary social support, constructing reasonable achievable career expectations, improving time management ability, and participating in psychological counselling programs.

**Supplementary Information:**

The online version contains supplementary material available at 10.1186/s12889-021-11544-8.

## Background

In China, village doctors refer to those who have obtained the “rural doctor qualification certificate” and work in village-level clinics, the most basic medical institutions in China, including those who have acquired the qualification of village-level practicing (assistant) doctors and those who have not obtained the above qualification [1]. The rural population accounts for 50.3% of China’s total population, still higher than the urban population, according to the precise data of China’s Sixth National Population Census [2]. Village doctors are “guardians” of rural residents’ health, responsible for providing all-round, multi-dimensional and sustainable basic services, covering prevention, treatment, health care, health education and management, etc. [3]. Therefore, as the most grass-roots and most widely existing medical service providers in rural areas, village doctors play an irreplaceable role in ensuring, protecting, and improving the health level of rural residents.

With the implementation of the new medical reform and the hierarchical medical system, the service mode of village doctors has changed from the passive “patient seeking medical treatment” to the active “health management service”, and from the combination of prevention and treatment to the comprehensive and whole-of-the-life-cycle health services [4]. The working mode of village doctors in village clinics is facing new and unprecedented challenges, they not only need to carry out daily medical treatment, but also public health, family doctor contract, family planning, chronic disease management and other services, which requires village doctors to update their knowledge and skills, and constantly improve the ability and quality of primary medical services [5]. However, in recent years, the situation of village doctors in China is facing severe challenges, including the difficulty of obtaining the qualification certificate of practicing doctors due to the low level of overall education, the poor level of wages and treatment, the difficulty of ensuring old-age insurance, the low social status, the difficulty of introducing young talents, and the overall instability of the ranks. The above problems seriously affect the survival of village doctors and the stability of the overall team. A survey of 2682 village doctors showed that village doctors had a low job satisfaction rate of 64.62%, especially in terms of income (24.98%), workload (39.34%), personal development (47.05%) and social status (50.34%) [6]. Poor satisfaction leads to high job burnout among village doctors, 68.60% of village doctors have moderate or above burnout, and 45.3% of village doctors have high turnover intention [7].

In 2019, there were two shocking and noticeable resignation incidents of village doctors, once 36 and once 28, in Henan province [5]. As a result, the total number of village doctors and the number of rural clinics in China is experiencing a sharp decline year by year. In May 2019, the statistical bulletin on the development of health undertakings showed that compared with 2017, China’s village doctors decreased by 62,000 in 2018, and the number of village clinics decreased by 5101. However, there are still 1022 administrative villages without clinics, 6903 clinics without qualified village doctors [8]. Thus, it can be seen that the job satisfaction, job burnout and turnover intention of village doctors have been related to the stability of the village doctors, closely related to the health level of farmers and the steady development of rural health undertakings, and have become the weakness and weakest link of China’s medical and health service system.

Because of its high incidence rate, job burnout is known as an epidemic of front-line medical staff [9], which is a universal and international hotspot that urgently needs attention in the field of occupational medical and health care. Maslach, in 2003, defined job burnout as a long-term, chronic response to work stress, and a persistent, harmful state syndrome related to work characteristics and working conditions [10]. However, the most widely accepted definition of burnout, defined by Maslach&Jackson, includes three components: emotional exhaustion (EE), depersonalization (DP), and low personal achievement (PA), also known as three-dimensional burnout syndrome. Among them, EE refers to a person who often shows a state of pessimism, depression, helplessness, hopelessness, and depression at work; DP refers to a person who often treats patients or colleagues with pessimistic, disgustful, negative attitude and indifferent emotion at work; and PA refers to a person’s slow decline in meaning and evaluation of the work performed and the feeling of being incompetent [11]. By the World Health Organization (WHO) in June 2019, burnout was included in its International Classification of Diseases Manual and defined as a syndrome caused by the failure of successful management of long-term job stress [12], and it was noted that if patients showed three symptoms, doctors could give the diagnosis of burnout: fatigue, cynicism about work, and difficulty in successfully completing work. This means job burnout has become an international occupational health problem and needs to attract people’s attention.

Job burnout reflects a long-term poor match between workers and their daily work, which is specifically demonstrated as a negative, powerless, and passive response to work pressure [13]. Job burnout beyond the warning usually causes negative and lazy self-image and negative psychological state of anxiety and depression, which leads to low attendance rate and low efficiency of doctors, and reduces the communication time between doctors and patients, which will lead to the decline of the quantity and quality of medical services [14], as well as the increase of the incidence of medical disputes and risks. When job burnout cannot be well treated and intervened, it may force doctors to transfer or leave the doctor’s post. If accumulated over a long period of time, it will have a negative impact on doctor’s psychosomatic and social interaction. Therefore, doctor’s job burnout, as the focus of occupational medical health, is a great subject related to people’s health and happiness [15].

Current researches suggest that the causes of doctors’ job burnout stem from three dimensions: social system, organization, and individual factors. Firstly, social factors include lack of social support, strict and cumbersome laws and regulations, aging, lack of social security, excessive income gap, patient satisfaction [16], etc. Secondly, organizational factors include management style, excessive workload, type of occupation, organizational change, inappropriate working conditions, endless overtime, poor organizational cohesion and low efficiency, insufficient career development opportunities, fierce competition, lack of organizational belonging, and excessive working hours each time. Finally, individual factors include: sex, age, relationship between husband and wife, whether there are young children, role conflicts, personality, self-esteem, ways to deal with difficulties etc. [14, 17–19]. However, the influence of personality and alexithymia on burnout of village doctors has not been studied. At the same time, although the study believes that work engagement is an effective measure to interfere with job burnout of village doctors [15], the impact of work engagement on burnout is still lack of empirical research. Meanwhile, existing studies have mostly used chi-square test, t-test, Analysis of Variance (ANOVA) and, multiple linear or multiple logistic regression analysis, to analyze the factors influencing job burnout [14, 19–25]. Compared with the above research, structural equation modeling (SEM) can not only measure the correlation between research variables, but also systematically and deeply dig the correlation between potential variables, and even explain the causal relationship between variables. Therefore, the introduction of SEM to study the quantitative regression relationship can make up for the limitations of current research to some extent [26, 27].

Although job burnout comes from three aspects, when facing the same organizational background and social factors, not all people will suffer from burnout, which is related to the individual differences of burnout. On the aspect of individual factors, Swider and Zimmerman believed that personality, the sum of characteristics such as temperament, ability and character, was closely related to burnout and turnover, and basing on modern personality theory, they assumed that individuals’ personality can influence their cognition and response to the environment [28]. For example, when team members conflict, individuals with neuroticism may evaluate the conflict differently than other participants.

Many researches usually adopt the famous personality model proposed by Costa and McCrae [10], namely five factor models, which defines five groups of interdependent personality traits: neuroticism, extroversion, openness, easygoing and conscientiousness. Neuroticism reflects a person’s emotional instability and fear, people with high scores are usually worried, unhappy, and insecure; Extroversion reflects how confident, active, and talkative a person is and the higher the score, the better the level of social interaction. People with open personalities tend to be more curious and playful, preferring innovative and unconventional ideas; Agreeableness represent the individual’s orientation to others’ experience, interest, and goal, the higher the score is, the more modest, kind, and compassionate; Conscientiousness refers to a person with a cautious attitude to guide their daily behavior, therefore, serious people are often reliable and prudent. Many empirical studies have found that personality is negatively correlated with burnout and affects it directly and indirectly. Okan Taycan et al. through a survey of 139 doctors in semi-urban and rural areas in Turkey, found that their level of job burnout was slightly higher than that of urban doctors, and the neuroticism dimension in personality was a significant predictor of burnout [29]. Doctors with personality characteristics such as high hostility, introversion, neuroticism, aimlessness, hostility, and unwillingness to accept new things had higher occupational burnout according to Cheng-Chieh Lin et al. found in a study of 2230 doctors in Taiwan [23]. Further research found that neuroticism, agreeableness, openness, and friendliness were found to be predictors of emotional exhaustion, and individual achievement was determined by neuroticism, openness, conscientiousness, and hostility [30]. Personality can influence job burnout through the mediating effect of subjective well-being. Individuals with personality traits such as emotional instability, neuroticism, anxiety, and irritability had lower levels of happiness and higher levels of emotional burnout [31].

As a multi-dimensional personality structure, alexithymia represents a defect in emotional cognitive processing, which is frequently associated with major depression and anxiety disorders [32]. Even some researchers suggest that alexithymia secondary to depression, and is a manifestation of “state dependence.” [33]. Many researches indicated that people with alexithymia are more likely to develop the feeling of tension when staying in a high-pressure working environment, which may lead to the heavier emotional exhaustion, depression and depersonalization [32, 34], and even physiological diseases such as gastroenterology and hepatology disorders [35]. But no matter in the field of psychology or occupational health, few researches focus on the relationship and mechanism between burnout and alexithymia. In a few studies, A study of 95 nurses in Greece by Aikaterini Moulou et al. found that alexithymia was positively associated with depersonalization and emotional exhaustion in burnout and could influence overall burnout scores through personal achievement and family support. After investigating 159 nursing assistants in 10 nursing homes in northern Spain, Erkuden Aldaz et al. found that, after strictly controlling the mixed effect of work characteristics, alexithymia made a moderate contribution to the depersonalization and personal accomplishment dimensions of burnout [36].

A high level of work engagement is the key to high quality job performance. Work engagement is defined as a positive, fulfilling, work-related state of mind including three dimensions of vigor, dedication, and absorption [37]. Tatenda S. Mhlanga et al. believe that although researchers have now found many beneficial and positive consequences of work engagement, such as, improving work efficiency, however, few people know the multiple antecedents that lead to work engagement, in which personality is an important antecedent. A study covering 1236 nurses showed that neuroticism was negatively correlated with work engagement, thus, extroversion, openness, easygoing and conscientiousness were positively correlated with work engagement [38]. At the same time, a Chilean study found that people with higher personality scores tend to have higher work engagement, and such people gain a lower level of job burnout even when working under high pressure.

**Fig. 1:**
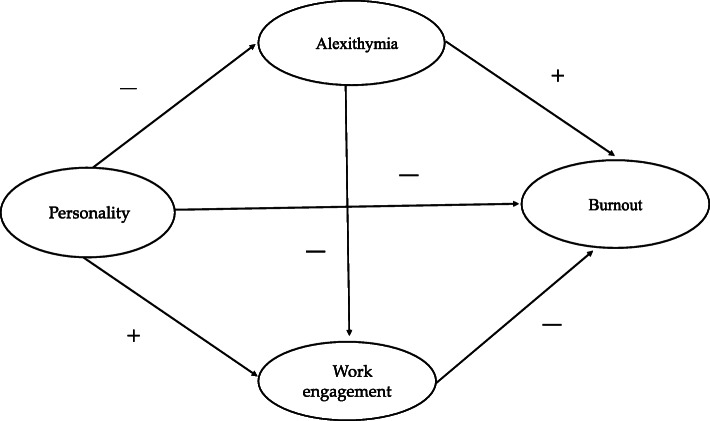
The theoretical model and hypotheses

After a comprehensive analysis of the above-mentioned theories and literature conclusions, the research is the first attempt to propose assumptions among personality, work engagement, alexithymia and job burnout and construct a double mediation model presented in Table 1 and Fig. 1. We hypothesize that village doctors’ personality, work engagement and alexithymia could have direct effects on burnout. Furthermore, personality could influence indirectly on the village medical staff’s burnout through work engagement and alexithymia.

**Table 1:** The Theoretical Hypotheses

Hypotheses	
1. Village doctors’ work engagement has a negative impact on burnout.	
2. Village doctors’ alexithymia has a positive impact on burnout.	
3. Village doctors’ personality has a positive impact on work engagement.	
4. Village doctors’ Personality has a negative impact on alexithymia.	
5. Village doctors’ Personality has a direct negative impact on burnout.	
6. Village doctors’ Personality has an indirect negative impact on burnout through the mediating effect of work engagement.	
7. Village doctors’ personality has an indirect negative impact on burnout through the mediating effect of alexithymia.

## Methods

### Setting and participants

Located in the southwest inland of Shandong Province, Jining lags behind the eastern coastal cities in economic development. In 2019, the total population of the city was 8.356 million, including 3.3683 million in rural areas. There were 6489 villages, with 5307 village clinics. At the end of 2018, the total number of medical personnel in the city reached 80,800, an increase of 2100 compared with 2017, but there were only 11,715 village doctors, 870 fewer than 2017 [39]. The average number of village doctors per village was only 1.81. However, the number of diagnosis and treatment in village clinics was far more than 21.26 million, with an average of 4006.41 per village clinic and 1814.94 per village doctor. Due to the heavy workload of village doctors, it is of great significance to take Jining city as the research site to understand the burnout status of village doctors and its influencing factors.

According to the method of stratified cluster random sampling, a designed cross-sectional study was carried out in the rural clinic of Jining City. First, according to the level of economic development, the 11 counties (districts) under the jurisdiction of Jining City were divided into three levels: better, medium and poor. Secondly, we randomly selected a county (district) as our sample source area in each level, and then all village doctors in the selected county (district) were taken as our research objects.

### Ethics and consent to participate

Our research group submitted an application for ethical review to the Institutional Review Board (IRB) of Jining Medical University. Based on the principle of informed consent and privacy protection, the expert group believed that this study did not involve medical intervention and human biological samples, and the individual cannot be tracked according to the research data. Therefore, the expert group of IRB approved that this study can collect data based on the oral consent of the participant. Before the on-site questionnaire survey, each participant was told that his participation was voluntary and anonymous and he / she had the right to refuse to participate and to terminate at any time during the course of the investigation. Questionnaires filled out by respondents who withdrew from the survey were destroyed in front of them.

Since the respondents were village doctors with a certain level of education and reading comprehension ability, the data were collected through the on-site self-filled questionnaire, including cover letter, personal general sociological characteristics, and research scales. In order to reduce the bias of the survey, we explained the significance of the research in the cover letter, as well as the anonymous survey did not involve personal information, thus increasing the response rate. Secondly, we set special personnel at each level of county, township, and village to manage and verify the questionnaire, so as to ensure the quantity and quality of the questionnaire. 2789 village doctors from 1345 rural clinics were surveyed in this study. Among them, 2693 questionnaires were returned, However, some of the survey subjects lacked too much filling in the scale related to this study, so 2684 questionnaires were valid in the end, with an effective rate of 96.2%.

### Measures

According to the Questionnaire on Human Resources for Health of the National Health Commission [40], the subject part of the questionnaire consists of five parts, in the first part, we designed relevant questions including the demographic characteristics (age, sex, marital status, level of education) and job characteristics (working years, major, salary, title, average working hours per week) of village doctors. Questions related to burnout, personality, alexithymia, and work engagement were in the second to fifth parts of the questionnaire.

#### Personality

The international Big Five Inventory (BFI) was used to evaluate village doctors’ personality traits. The reliability and validity of the big five scale were tested in different populations. Among them, the Cronbach’s alpha coefficient of the five dimensions measured in the Chinese population were 0.770 for extraversion, 0.694 for agreeableness, 0.737 for conscientiousness, 0.766 for neuroticism, and 0.738 for openness [41]. The whole set of big five inventory contains 44 questions, 5 dimensions, neuroticism (8 items), extroversion (8 items), openness (10 items), agreeableness (9 items), conscientiousness (9 items). Using a 5-point Likert scale ranging from 1 (totally disagree) to 5 (totally agree) to calculate and evaluate all these items, and some of which were scored in reverse. The higher the individual dimension scores, the stronger the personality traits of village doctors.

#### Alexithymia

Using the 20-Item Toronto Alexithymia Scale (TAS-20) to measure the alexithymia status of village doctors. With a Cronbach’s alpha value of 0.87 [42], the scale was verified to have a good reliability and validity in the Chinese population and consists of 3 subscale scales for identifying difficulties, describing emotional difficulties and extroverted thinking difficulties. A 5-point Likert scale, from 1(total disagreement) to 5(fully agree), was used to assess items, of which 5 items were negative. The total scores of 20 items ranged from 20 to 100, the higher the score, the more serious the level of alexithymia. Basing on experience, the researchers determined cutoff scores, and greater than 60 points were highly alexithymia and less than 52 points were lack of alexithymia [43].

#### Work engagement

The current status of work engagement of Chinese village doctors was measured by Utrecht Work Engagement Scale (UWES) with 17 items. The Cronbach’s alpha value of Chinese version of the scale was 0.782 [7]. The whole scale was divided into three dimensions, including 6 items for work absorption and 6 items for work vigour, and 5 items for work dedication. Each item was measured and scored though a 7-point Likert scale ranging from 0 (never) to 6 (always). The higher the score is, the higher the enthusiasm of village doctors for rural medical work [44].

#### Burnout

The Chinese version of Maslach Burnout Scale General Scale (MBI-GS), with a Cronbach’s alpha ranging from 0.79 to 0.94 [45, 46], was used to measure the burnout of village doctors [47]. MBI has three subscales, including emotional exhaustion, depersonalization, and low personal achievement (reverse score). The answers for items were 7 Likert score ranging from 0 (never) to 6 (daily). Among them, the higher the score of emotional exhaustion, depersonalization, and low personal achievement (reverse score), the heavier the degree of burnout. That is, in terms of emotional exhaustion, points less than 17 and greater than 25 are defined as low burnout and high burnout, between 17 and 25 points are defined as moderate burnout; In terms of depersonalization, scores less than 7 and greater than 11 are defined as low burnout and high burnout, and scores between 7 and 11 are defined as moderate burnout; In terms of low personal achievement, scores less than 12 and greater than 16 are defined as low burnout and high burnout, and scores between 12 and 16 are defined as moderate burnout. When judging the overall burnout level of occupational burnout, the critical scores of the three dimensions are 25, 11, and 16, respectively. Burnout can be diagnosed when the score of any one dimension of the survey subject’s score is greater than the critical value [44].

### Statistical analysis

Exploratory Factor Analysis (EFA), an important tool for multivariate data analysis, was used to scientifically evaluate the reliability and validity of the questionnaire in this study. The socio-demographic and work characteristics of 2684 village doctors were described by descriptive statistics. Then, a descriptive analysis of personality, alexithymia, work engagement and job burnout were conducted and the results were presented as mean and standard deviation (SD). Correlation between the values of the main observed variables was measured by Pearson correlation and quantified by correlation coefficient. On the basis of the above research results, using the structural equation model (SEM), we further explored and quantified the relationship between the four dimensions of personality, alexithymia, work engagement and job burnout, and applied the bootstrap-based maximum likelihood model in the SEM. Several key metrics to measure model fit with data, including normed fit index (NFI), goodness of fit index (GFI), comparative fit index (CFI), adjusted goodness of fit index (AGFI), Tucker-Lewis index (TLI) and incremental (IFI) were all greater than 0.90, while root mean square error of approximation (RMSEA) was 0.078, below 0.8, reflecting that this was an acceptable model consistent with the fit between current data and assumptions.

### Reliability and validity

The results of Exploratory factor analysis (EFA) showed that the KMO (Kaier-Meyer-Olkin) of the whole questionnaire was 0.825, greater than 0.70, according to the requirements of factor analysis. Bartlett test of sphericity was also significant (^2^ = 23,750.004, *P* < 0.001). In the factor load analysis, the maximum coefficient of variation method was used for orthogonal rotation (varimax), and the result of factor load matrix after rotation was obtained as follows: The characteristic roots of the four evaluation indexes were all significantly more than 1, and the contribution rate of the accumulated variance was 80.092%. The load value of each item in the corresponding dimension was greater than 0.758, proving the structural validity of the whole questionnaire was good. Meanwhile, the Cronbach’s α of the whole questionnaire was 0.741, indicating good reliability of internal consistency [7].

## Results

### Demographic and working characteristics of participants

Among the 2864 village doctors, their mean age was 44.64 ± 7.248 years, the maximum age was 87, and the minimum age was 21; Male doctors accounted for 64.4%, and age stratification showed that only 1.3% were under 30 years of age and only 2.6% were university and above; As many as 62.4% of village doctors failed to obtain the village doctors’ professional qualification, only 3.8% of them had middle and senior professional titles. Nearly half of them earned less than 2000 yuan a month, but nearly 70% worked far more than 60 h a week, as shown in Table 2.

**Table 2:** Demographic characteristics of participants (*n* = 2693)

Socio–Demographic	N	%
**Sex**		
Male	1729	64.4
Female	920	34.3
Missing	35	1.3
**Age, Group**		
< 30 years	35	1.3
30–39 years	622	23.2
40–49 years	1298	48.4
≥ 50 years	683	25.4
Missing	46	1.7
**Level of education**		
University or above	70	2.6
Junior College	656	24.4
Technical secondary school	1830	68.2
High school education or below	91	3.4
Missing	37	1.4
**Practicing requirements**		
Rural general practitioner	334	13
Rural assistant physician	442	17.2
Chinese medicine assistant physician	192	7.5
Unqualified	1607	62.4
Missing	109	4.1
**Professional ranks**		
Middle or high profession	101	3.8
Primary title	1306	48.7
No title	1157	43.1
Missing	120	4.5
**Monthly income (yuan)**		
< 2000	1247	46.5
2000–2999	767	28.6
≥ 3000	505	18.8
Missing	165	6.1
**Weekly working hours**		
< 40	388	14.5
40–59	336	12.5
≥ 60	1875	69.9
Missing	85	3.2

### Quantitative descriptive analysis of main variables

The total scores of job burnout, personality, alexithymia, and work engagement of village doctors were 42.46 ± 21.099, 145.71 ± 13.002, 55.11 ± 9.714, 66.15 ± 20.255 respectively. The scores for each dimension of each variable are detailed in Table 3.

**Table 3:** Item scores in burnout, personality, alexithymia, and work engagement

Items	Mean ± SD
**Burnout**	**42.46 ± 21.099**
Emotional exhaustion	18.97 ± 12.281
Low personal achievement	17.53 ± 13.419
Depersonalization	5.96 ± 6.913
**Personality**	**145.71 ± 13.002**
Neuroticism	26.30 ± 4.338
Extraversion	26.04 ± 3.879
Openness	32.37 ± 4.826
Agreeableness	33.22 ± 5.189
Consciousness	32.53 ± 5.252
**Alexithymia**	**55.11 ± 9.714**
Difficulty describing feelings	14.21 ± 2.836
Difficulty recognizing feelings	19.25 ± 5.427
Externally oriented thinking	22.36 ± 18.758
**Work engagement**	**66.15 ± 20.255**
Work vigor	23.57 ± 7.023
Work dedication	19.70 ± 6.280
Work absorption	22.88 ± 7.628

According to the results, 1762 (65.2%) of the 2693 village doctors were diagnosed with burnout according to the burnout assessment criteria. Among the three dimensions, 811(30.1%) of the village doctors surveyed had severe emotional exhaustion, 652(24.2%) had moderate emotional exhaustion, 1230 (45.7%) had low emotional exhaustion. 1300(48.3%) of the village doctors had severe low personal achievement, 359(13.3%) had moderate low personal achievement, and 1034(38.4%) had low personal achievement; 599(22.2%) of the village doctors had severe depersonalization burnout, 316(11.7%) had moderate depersonalization burnout, and 1778(66.0%) had low depersonalization burnout. Meanwhile, 1112(41.4) of village doctors had suspected alexithymia and 679(25.3%) had apparent alexithymia. Please refer to Table 3 for details.

### Correlations of study variables

The main observed variables’ Pearson correlation coefficients were shown in Table 4. Personality was significantly negatively correlated with burnout and alexithymia, and positively correlated with work engagement, which was significantly negatively correlated with alexithymia and burnout. Finally, alexithymia was positively correlated with burnout.

**Table 4:** Correlation coefficients among study variables

Items	Personality	Alexithymia	Work engagement	Burnout
Personality				
Alexithymia	−.0308**			
Work engagement	0.450**	−0.265**		
Burnout	−0.380**	0.512**	−0.389**	

### Testing of the constructed study model

**Fig. 2:**
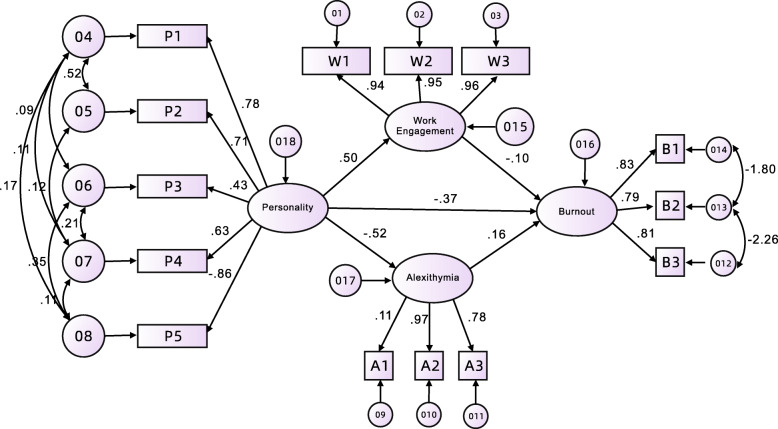
The final model and standardised model paths

The SEM was built to connect, test, and evaluate the interrelationships among the four variables (burnout, personality, alexithymia, and work engagement). The accurate generalized least square method based on the optimization model was used to fit the data and the theoretical model, and the theoretical model was modified and improved according to the fitting results. The final fit-corrected model (Fig. 2) shows the interrelation, valid path, and effect values between the four variables. The final modified hypothesis model fit index indices were AGFI = 0.923, GFI = 0.934, NFI = 0.946, CFI = 0.948, IFI = 0.948, TLI = 0.924, RMSEA = 0.078, all conformed to the reference values given by acceptable model fit.

Using maximum likelihood estimation, each path was guided by the 200 repetitions of bias-corrected bootstrap, mediation analysis path as well as effect values were shown in Table 5. Personality had a direct positive effect on work engagement (β = 0.50, *p* < 0.001), but had a direct negative effect on alexithymia (β = − 0.52, p < 0.001) and burnout (β = − 0.50, p < 0.001) respectively. Work engagement had a direct negative effect on burnout (β = − 0.10, p < 0.001), while alexithymia had a direct and significant positive effect on burnout (β = 0.16, p < 0.001).

**Table 5:** Significance test of the mediating test

Model Pathways	Estimated	95% CI
**Total effects**		
Work engagement ← Personality	0.50	0.46–0.54
Alexithymia← Personality	− 0.52	(− 0.56) - (− 0.48)
Burnout ←Personality	− 0.50	(− 0.54) - (− 0.47)
Burnout ← Work engagement	− 0.10	(− 0.14) - (− 0.06)
Burnout ← Alexithymia	0.16	0.12–0.21
**Direct effects**		
Work engagement ← Personality	0.50	0.46–0.54
Alexithymia← Personality	− 0.52	(− 0.56) - (− 0.48)
Burnout ←Personality	− 0.37	(− 0.42) - (− 0.32)
Burnout ← Work engagement	− 0.10	(− 0.13) - (− 0.06)
Burnout ← Alexithymia	0.16	0.11–0.21
**Indirect effects**		
Burnout ←Personality	− 0.13	(− 0.17) - (− 0.10)

Table 6 displays the significant test results of the two mediated pathways. In the intermediary path between personality and burnout, both alexithymia 95%CI:(− 0.36)–(− 0.09), and work engagement 95%CI:(− 0.17)–(− 0.08), had significant mediating effects.

**Table 6:** Significance test of every mediating pathway

Model Pathways 95% CI	95% CI
Burnout ← Work engagement ← Personality	(− 0.17) – (− 0.08)
Burnout ← Alexithymia← Personality	(−0.36) – (− 0.09)

## Discussion

The purpose of this study was to explore the serious situation of burnout of village doctors at the grass-roots level in China, and the alexithymia, personality, and work engagement on the impact of burnout. The important unique value of this study lies not only in the selection of Chinese village doctors, the most basic doctors, as our research subjects, but also in the incorporation of these four variables into the structural model for the first time.

The results showed that 65.2% of the village doctors were diagnosed with burnout, and in the three dimensions of job burnout, the proportion of village doctors’ low personal achievement was higher than that of the other two dimensions, there were 1300(48.3%) village doctors with a serious low personal achievement, meanwhile, 811 (30.1%) with severe emotional exhaustion, 599(22.2%) with severe depersonalization, which is similar to the findings of Lin Li et al. ‘s survey on 759 village doctors in southern China, which showed that 88.67% of them had a serious low personal achievement, 24.38% had severe emotional exhaustion, and 12.12% had severe depersonalization [47]. Compared with other doctors in other high-level hospitals, the burnout of village doctors was more serious and should be concerned. A cross-sectional study by Hui Wu et al. of 1202 specialists in affiliated hospitals of medical universities in China found that the high degree of burnout in the three dimensions accounted for 12.1% of the surveyed doctors [48]. And a recent meta-analysis by Carolina s. et al. found that low achievement was the most important factor affecting nurses’ burnout in primary health care Settings, accounting for 31% of the sample, followed by emotional exhaustion (28%), and depersonalization (15%) [24]. Thus, as medical staff of primary health services, the ranking of the effects of the three dimensions of job burnout among village doctors in our study sample was similar to that of the meta-analysis, but the positive rate of each dimension was much higher than that of primary nurses in this meta-analysis.

According to the American Physician Burnout and Depression Report (2018), doctors aged 45 to 54 experienced the highest burnout among all working age groups, at 50%. Similarly, Sun’s survey of 359 village doctors in Anhui province, China found that village doctors in the 46 to 55 age group had the highest scores for burnout and emotional exhaustion [49]. People aged 40–49 accounted for almost half of the total number of village doctors in our survey, while people under 30 accounted for only 1.3%, so we hypothesized that age was also a reason for the high burnout among village doctors. Only 2.6% of village doctors had a college degree or above, only 37.7% had a village (assistant) doctor qualification, and less than 4% had an intermediate title or above. Although the national policies have reduced the difficulty of the vocational qualification examination for village doctors in recent years, many village doctors still cannot pass the examination due to the limitations of education and technical level, and limits the promotion of vocational qualification for village doctors. Which in turn make rural residents lack trust in the medical work of village doctors, and doctor-patient disputes often occur [50]. Meanwhile, they had a heavy workload, of which 69.9% need to work 60 h or more, however, their income was below the normal salary level, with 46.5% of them earning less than 2000 yuan a month. All these factors lead to severe burnout among existing doctors. Therefore, in order to alleviate the phenomenon of village doctors’ job burnout in China, more research is urgently needed to explore the key factors of village doctors’ burnout and its influencing mechanism.

The equation model proved that personality affect the burnout of village doctors through direct and indirect paths. At present, the research objects of the relationship between personality and burnout of medical staff mostly focused on nurses in pediatrics, oncology department, emergency diagnosis and serious illness department, and the research on doctors, especially the primary doctors, is very rare. A cross-sectional study of 1357 nurses found that job burnout in nurses was negatively correlated with extroversion, agreeableness, conscientiousness and openness, and positively correlated with neuroticism [51]. A meta-analysis of the relationship between nurses’ personality and burnout found that the personality characteristics of the five-factor model could explain the significant differences in each burnout dimension [52]. A further study found that neuroticism was the most important personality trait to affect job burnout, while conscientiousness and neuroticism were the most significant personality traits to predict job burnout [53]. Although these studies focused on the influence of personality on burnout, the methods used were mostly limited to descriptive statistics, Pearson correlation analysis and hierarchical linear regression analysis to evaluate the linear relationship, and few mediating factors were introduced to measure the indirect influence of personality on burnout [53, 54].

The model also proved that work engagement plays a mediating role between personality and job burnout, though the relationship between burnout and work engagement is still controversial in academic circles [55]. While some researchers propose that burnout is both the opposite and an extreme concept of work engagement [56]. A study of 4457 British medical staff found that doctors’ burnout was associated with high neuroticism in personality, while their work engagement was closely related to extroversion and consciousness in personality, and doctors with high levels of participation still suffered from burnout [57]. Although some relevant studies have shown the weak relationship between work engagement and personality, burnout, but there is still a lack of quantitative analysis especially the intermediary exploration, between these factors, and our research can make up for this limitation. Through correlation analysis, we found that work engagement of village doctors was negatively correlated with alexithymia and burnout, and positively correlated with personality. Through in-depth SEM analysis, we found that work engagement of village doctors not only directly had a negative impact on burnout, but also played a significant mediating role between personality and burnout of village doctors.

Known as “emotional dysphoria”, alexithymia is not an independent disease, but a personality trait. So far, there are relatively few studies on the relationship between alexithymia and job burnout in academic circles, especially quantitative studies. Our finding, more than a quarter of village doctors have obvious alexithymia, which showed a direct positive effect of alexithymia on burnout, was similar to a Finnish study of 3322 employees aged 30–64 years, which showed that alexithymia was highly likely to be an independent risk factor for burnout after controlling for possible confounding factors [32]. Our in-depth SEM study indicated that alexithymia can also mediate the relationship between personality and burnout. D Lazzari’s study of 238 medical workers also found that alexithymia played an intermediary role in organizational environment and burnout. Depersonalization in burnout was the main influencing factor of alexithymia, so the concept of alexithymia should be included in the prediction model of various dimensions of job burnout [58].

To sum up, our study used structural equations to reveal the three influencing pathways of village doctors’ job burnout. Personality, work engagement and alexithymia were all accurate predictors of village doctors’ job burnout. The results further verified the complex influence of village doctors’ personality on their burnout. The reason was that personality can not only have a direct positive effect on burnout, but also indirectly affect burnout through the intermediary effect of work engagement and alexithymia. So, hence, this suggests may be a more sophisticated and in-depth mechanism in the relationship between personality and burnout. However, there is still a lack of research on the burnout of village doctors. In the future, we should not only pay attention to the factors that lead to burnout, but also study effective intervention measures. From the perspective of predictive factors, we should construct perfect intervention measures for village doctors’ work status, so as to reduce burnout and increase work engagement. In addition, as job burnout is a subjective and complex multi-dimensional variable, it is difficult to analyze it in detail through quantitative research. Qualitative research method should be used to explore the burnout of village doctors in micro and deep level in the future.

Three limitations of the study should be addressed. First of all, although this study used SEM to verify the relationship between variables, at the beginning of the study, factors as “cause”, including work engagement, personality and alexithymia, and the factors as “effect”, burnout, existed at the same time, so it was impossible to determine the sequence of occurrence among the factors, and there was no control group for comparison. Therefore, it cannot determine the causal relationship between the factors and draw a clear conclusion based on the cross-sectional design. Secondly, we collected data by self-filling rather than face to face investigation. Thirdly, since there is no unified standard for the diagnosis of burnout, some scholars believed that burnout was considered as long as any of the three dimensions in the MBI scale was above the critical value [43]. Some argued that two [59] or even three dimensions [60] must be above the critical value to determine burnout. And our study adopted the first diagnostic, which may increase the detection rate of burnout.

## Conclusions

Doctors’ job burnout is costly for hospitals (clinics), individuals, and patients. Therefore, it is the responsibility of organizations and individuals to ensure a high degree of “fit” between work and individuals. It is very important to control and cultivate the personality traits of medical students in the education and training stage. At the same time, it is also very important for doctors to have a professional psychological test of their personality traits before they enter the work force. To ensure the accuracy of personality testing, it is important to establish the reliability and validity of personality testing tools suitable for village doctors, and to ensure that the effects of personality in different situations are consistent. The Big Five can meet this requirement. More than a quarter of village doctors in our study have obvious alexithymia, which can not only have a direct positive effect on burnout, but also play a mediating role between personality and burnout. Many studies have also shown that alexithymia is associated with neuroticism in personality, which is the definitive cause of burnout. Therefore, with the increasing demand for medical care by rural residents, village doctors have a strong work intensity, low pay, insecure old-age care, and occupational pressure is also increasing. Village doctors’ alexithymia characteristics and psychological conditions have changed greatly, becoming an early warning and intermediary factor of burnout.

Based on the above research conclusions, the following improvement suggestions were put forward. First, because the personality characteristics of village doctors are closely related to burnout, and medical administrators should pay attention to the personality characteristics of village doctors in their professional training, practice selection and job assignment, so as to improve the medical quality and reduce the level of job burnout. Meanwhile, to encourage doctors to actively reflect on their own personality which causes job burnout and stress, and to improve their personal personality characteristics and reduce job burnout by obtaining necessary family and social support, constructing reasonable and feasible career expectations, improving time management skills and actively participating in psychological counseling programs [61]. Secondly, in view of the positive effect of neuroticism on burnout, it is suggested that village doctors should learn to control their emotions in their daily work life, at the same time learn to talk, but also learn to divert attention. Trying to distract oneself when one’s emotions are out of control is the best way to control one’s emotions and reduce burnout. Thirdly, the early identification of alexithymia in village doctors is helpful to predict the degree of personality improvement and to implement psychological intervention as soon as possible. These can reduce job burnout and improve the quality of medical services.

## Supplementary Information

**Additional file 1.** STROBE_checklist_v4_combined_PlosMedicine.

**Additional file 2.** Questionnaire on the current situation of burnout among village doctors and its relationship with personality, alexithymia, and work engagement.

## Data Availability

The datasets used and/or analyzed during the current study are available from the corresponding author on reasonable request.

## Abbreviations

TAS-20: 20-Item Toronto Alexithymia Scale
BFI: Big Five Inventory
SD: Standard Deviation
EFA: exploratory factor analysis
SEM: Structural Equation Modeling
AGFI: adjust goodness of fit index
NFI: normed fit index
GFI: goodness of fit index
CFI: comparative fit index
TLI: Tucker-Lewis index
IFI: incremental
RMSEA: root mean square error of approximation
ANOVA: Analysis of Variance
IRB: Institutional Review Board
