# Supplementary material for: The influence of personality, alexithymia and work engagement on burnout among village doctors in China: a cross-sectional study
Source: BMC Public Health. 2021 Aug 4;21:1507. doi: 10.1186/s12889-021-11544-8 (PMC8335472; doi:10.1186/s12889-021-11544-8)
Supplement: Supplementary file 2 — Additional file 2. Questionnaire on the current situation of burnout among village doctors and its relationship with personality, alexithymia, and work engagement. [file 12889_2021_11544_MOESM2_ESM.docx]

**Questionnaire on the current situation of burnout among village doctors and its relationship with personality alexithymia and work engagement.**

Dear fellow doctors’ friends: Hello! In order to understand the current situation of rural medical staff, in order to better maintain the physical and mental health of rural medical personnel, you are invited to participate in this survey. The credibility of your research results depends on your serious and objective answers to the questions, please fill out this questionnaire, carefully read the questions, and truly express your feelings. This survey does not remember the name, the answer is not right or wrong, we will be strictly confidential survey information, please do not have any concerns. Thank you for your support and cooperation.

**Table 1** **Demographic and working characteristics of participants**(Please **√** under the serial number that matches your situation)

| 1 | Sex: (1) Male (2) Female | 2 | Age: |
| --- | --- | --- | --- |
| 3 | Your current marital status is: (1) not married (including unmarried, divorced, and widowed) (2) married | | |
| 4 | Your highest education is: (1) Undergraduate and above (2) post-secondary (3) High School, Secondary and Technical School (4) Junior High School (5) Primary School and below | | |
| 5 | The unit you are working in belongs to:(1) Village Health Room (2) Private clinic (multi-person joint) (3) Private clinic (personally run). | | |
| 6 | Your current title is: (1) Advanced (2) Intermediate (3) Division (Primary) (4) medical assistant (5) No Title | | |
| 7 | Your current qualifications are: (1) Practicing Physician (2) Practicing Assistant Physician (3) Chinese Medicine Practitioner (4) Chinese Medicine Practitioner (5) Registered Nurse (6) Other | | |
| 8 | You are engaged in medical work for approximately | 16 | You are working in your current unit for approximately |
| 9 | Last year, your average monthly income was approximately RMB (including wages, bonuses, subsidies, and all other income). | | |
| 10 | Have you been promoted to your title in the past 5 years? (1) Yes (2) No | | |
| 11 | Your average working week is approximately | | |
| 12 | On average, you work night shifts per unit per month (excluding home listening classes) approximately | | |

**Table 2** **Work engagement** (Please **√** on the option that best suits your situation, depending on your actual feelings)

| **project** | | **Never** | **Almost none** | **seldom** | **sometimes** | **often** | **Very often** | **always** |
| --- | --- | --- | --- | --- | --- | --- | --- | --- |
| 1 | At work, I feel like I'm emitting energy |  |  |  |  |  |  |  |
| 2 | I feel that the purpose of the work I do is clear and meaningful |  |  |  |  |  |  |  |
| 3 | When I work, time always goes by fast |  |  |  |  |  |  |  |
| 4 | At work, I feel strong and energetic |  |  |  |  |  |  |  |
| 5 | I am passionate about my work |  |  |  |  |  |  |  |
| 6 | I forget everything around me when I work |  |  |  |  |  |  |  |
| 7 | My work inspired me |  |  |  |  |  |  |  |
| 8 | As soon as I get up in the morning, I want to go to work |  |  |  |  |  |  |  |
| 9 | When I work nervously, I feel happy |  |  |  |  |  |  |  |
| 10 | I am proud and proud of what I have done |  |  |  |  |  |  |  |
| 11 | I'm immersed in my work |  |  |  |  |  |  |  |
| 12 | I can work continuously for a long time at a time |  |  |  |  |  |  |  |
| 13 | My job is challenging for me |  |  |  |  |  |  |  |
| 14 | I will reach the level of forgetfulness at work |  |  |  |  |  |  |  |
| 15 | At work, even if I feel mentally tired, I can recover quickly |  |  |  |  |  |  |  |
| 16 | I feel like I can't live without this job |  |  |  |  |  |  |  |
| 17 | Even if the work doesn't go well, I can always stick to it |  |  |  |  |  |  |  |

**Table 3 Burnout** (Please √ based on your true feelings at work on the appropriate option that best reflects how often you feel)

|  | | Never | Several times a year | Once a month | Several times a month | Once a week | Several times a week | Every day |
| --- | --- | --- | --- | --- | --- | --- | --- | --- |
| 1 | Medical work makes me depressed |  |  |  |  |  |  |  |
| 2 | I feel very tired after a day's work |  |  |  |  |  |  |  |
| 3 | When I got up in the morning, I felt very tired, but I still had to face the day's work |  |  |  |  |  |  |  |
| 4 | I can easily understand how the patient feels throughout the course of medical care |  |  |  |  |  |  |  |
| 5 | I sometimes think of patients as an object, not as a person |  |  |  |  |  |  |  |
| 6 | It's a burden for me to work with people all day long |  |  |  |  |  |  |  |
| 7 | I can effectively deal with the problems that arise during medical care |  |  |  |  |  |  |  |
| 8 | Medical work drained me of my heart |  |  |  |  |  |  |  |
| 9 | I feel that my work has played a positive role in the lives of others |  |  |  |  |  |  |  |
| 10 | After taking this job, I became more and more cold to others |  |  |  |  |  |  |  |
| 11 | I'm afraid this job will turn me into a hard-hearted person |  |  |  |  |  |  |  |
| 12 | I feel energetic |  |  |  |  |  |  |  |
| 13 | I feel that I have suffered a setback in my work |  |  |  |  |  |  |  |
| 14 | I feel that I work too hard |  |  |  |  |  |  |  |
| 15 | I don't pay much attention to the various needs put forward by patients |  |  |  |  |  |  |  |
| 16 | Working with other people puts me under a lot of pressure |  |  |  |  |  |  |  |
| 17 | When I get along with patients, I can easily create a relaxed atmosphere |  |  |  |  |  |  |  |
| 18 | Close contact with the patient at work makes me feel satisfied and happy |  |  |  |  |  |  |  |
| 19 | Doing medical work gives me the value of myself |  |  |  |  |  |  |  |
| 20 | I feel like I can't put more emotion into my work |  |  |  |  |  |  |  |
| 21 | I can calmly deal with the emotional distress I encounter at work |  |  |  |  |  |  |  |
| 22 | I think some patients blame me for their own problems |  |  |  |  |  |  |  |

**Table 4** **personality** (Please **√** on the option that best suits your situation, depending on your actual feelings)

| **project** | | **I totally disagree** | **disagree** | **So so** | **agree** | **I totally agree** |
| --- | --- | --- | --- | --- | --- | --- |
| 1 | I'm very talkative |  |  |  |  |  |
| 2 | I like to pick on other people's faults |  |  |  |  |  |
| 3 | I'm serious about my work |  |  |  |  |  |
| 4 | I often feel depressed and depressed |  |  |  |  |  |
| 5 | I'm original and often produce new ideas |  |  |  |  |  |
| 6 | My character is introvertedly silent |  |  |  |  |  |
| 7 | I am selfless and helpful |  |  |  |  |  |
| 8 | I may be a little careless |  |  |  |  |  |
| 9 | I often feel relaxed and able to cope well with the stresses of work and life |  |  |  |  |  |
| 10 | I'm curious about different things |  |  |  |  |  |
| 11 | I'm full of energy |  |  |  |  |  |
| 12 | I often quarrel with others |  |  |  |  |  |
| 13 | I'm a trustworthy person |  |  |  |  |  |
| 14 | I get nervous sometimes |  |  |  |  |  |
| 15 | I am original and deeply thought-out |  |  |  |  |  |
| 16 | I'm very enthusiastic |  |  |  |  |  |
| 17 | I am open-minded and cheerful |  |  |  |  |  |
| 18 | I don't do things in an orderly way |  |  |  |  |  |
| 19 | I worry too much about the present and the future |  |  |  |  |  |
| 20 | I imagine thinking actively |  |  |  |  |  |
| 21 | My personality rest |  |  |  |  |  |
| 22 | I trust others |  |  |  |  |  |
| 23 | I'm lazy |  |  |  |  |  |
| 24 | I'm emotionally stable and not easily restless |  |  |  |  |  |
| 25 | I'm creative |  |  |  |  |  |
| 26 | I have a decisive personality |  |  |  |  |  |
| 27 | My character may be cold and lonely |  |  |  |  |  |
| 28 | I persevered in my work until the task was finished |  |  |  |  |  |
| 29 | I have a moody personality |  |  |  |  |  |
| 30 | I value artistic and aesthetic experiences |  |  |  |  |  |
| 31 | My character is sometimes shy |  |  |  |  |  |
| 32 | I am reasonable and amiable to people |  |  |  |  |  |
| 33 | I do things efficiently |  |  |  |  |  |
| 34 | I can keep calm in tense situations |  |  |  |  |  |
| 35 | I like to do regular work; I don't like uncertain work |  |  |  |  |  |
| 36 | I'm outgoing and good at socializing |  |  |  |  |  |
| 37 | I am sometimes rude to others |  |  |  |  |  |
| 38 | I often make plans and go according to plan |  |  |  |  |  |
| 39 | I'm prone to nervousness |  |  |  |  |  |
| 40 | I'm good at thinking |  |  |  |  |  |
| 41 | I'm hardly interested in art |  |  |  |  |  |
| 42 | I prefer to work with others rather than compete |  |  |  |  |  |
| 43 | I'm easily distracted |  |  |  |  |  |
| 44 | I am good at art, music or literature |  |  |  |  |  |

**Table 5 Alexithymia** (Please **√** on the option that best suits your situation, depending on your actual feelings)

| **project** | | **I totally disagree** | **disagree** | **So so** | **agree** | **I totally agree** |
| --- | --- | --- | --- | --- | --- | --- |
| 1 | I often can't figure out how I feel inside |  |  |  |  |  |
| 2 | It's hard for me to describe how I feel in the right words |  |  |  |  |  |
| 3 | I have some physical sensations that even the doctor who sees me can't understand |  |  |  |  |  |
| 4 | I can easily describe how I feel |  |  |  |  |  |
| 5 | I prefer to analyze problems rather than just describe them |  |  |  |  |  |
| 6 | When I feel bad, I don't know if it's sadness, fear, or anger |  |  |  |  |  |
| 7 | I am often confused about some of the feelings in my body |  |  |  |  |  |
| 8 | I prefer to let things happen, rather than pursue the cause |  |  |  |  |  |
| 9 | I have feelings that I can't even recognize |  |  |  |  |  |
| 10 | I am very concerned about my inner experience |  |  |  |  |  |
| 11 | I can't describe how I feel about others |  |  |  |  |  |
| 12 | Others give me time and opportunity to describe my inner feelings |  |  |  |  |  |
| 13 | I don't know what kind of psychological activity is going on inside |  |  |  |  |  |
| 14 | I often don't know why I'm angry |  |  |  |  |  |
| 15 | I like to talk to others about his daily activities rather than his inner feelings |  |  |  |  |  |
| 16 | I prefer watching light-hearted entertainment movies to watching plot films about personal destiny |  |  |  |  |  |
| 17 | Even to close friends, I can't express my innermost feelings |  |  |  |  |  |
| 18 | I can feel intimacy with someone, even when we are silent |  |  |  |  |  |
| 19 | I find it useful to reflect on yourself to solve personal problems |  |  |  |  |  |
| 20 | Finding hidden meaning in movies and plays can be a distraction from entertainment |  |  |  |  |  |
